# Gender Differences in Associations of *Glutamate Decarboxylase 1* Gene (*GAD1*) Variants with Panic Disorder

**DOI:** 10.1371/journal.pone.0037651

**Published:** 2012-05-25

**Authors:** Heike Weber, Claus Jürgen Scholz, Katharina Domschke, Christian Baumann, Benedikt Klauke, Christian P. Jacob, Wolfgang Maier, Jürgen Fritze, Borwin Bandelow, Peter Michael Zwanzger, Thomas Lang, Lydia Fehm, Andreas Ströhle, Alfons Hamm, Alexander L. Gerlach, Georg W. Alpers, Tilo Kircher, Hans-Ulrich Wittchen, Volker Arolt, Paul Pauli, Jürgen Deckert, Andreas Reif

**Affiliations:** 1 Department of Psychiatry, Psychosomatics and Psychotherapy, University of Würzburg, Würzburg, Germany; 2 Interdisciplinary Center for Clinical Research (IZKF), University of Würzburg, Würzburg, Germany; 3 Department of Psychiatry and Psychotherapy, University of Münster, Münster, Germany; 4 Department of Psychiatry, University of Bonn, Bonn, Germany; 5 Department of Psychiatry, University of Frankfurt, Frankfurt, Germany; 6 Department of Psychiatry, University of Göttingen, Göttingen, Germany; 7 Christoph-Dornier-Foundation for Clinical Psychology, Bremen, Germany; 8 Zentrum für Klinische Psychologie und Rehabilitation, University of Bremen, Bremen, Germany; 9 Institute of Psychology, University of Berlin, Berlin, Germany; 10 Department of Psychiatry, Charité, Berlin, Germany; 11 Institute of Psychology, University of Greifswald, Greifswald, Germany; 12 Institute of Clinical Psychology and Psychotherapy, University of Köln, Köln, Germany; 13 Department of Psychology, University of Würzburg, Würzburg, Germany; 14 Clinical and Biological Psychology, School of Social Sciences, University of Mannheim, Mannheim, Germany; 15 Department of Psychiatry, University of Marburg, Marburg, Germany; 16 Institute of Clinical Psychology and Psychotherapy, Technical University of Dresden, Dresden, Germany; Chiba University Center for Forensic Mental Health, Japan

## Abstract

**Background:**

Panic disorder is common (5% prevalence) and females are twice as likely to be affected as males. The heritable component of panic disorder is estimated at 48%. Glutamic acid dehydrogenase GAD1, the key enzyme for the synthesis of the inhibitory and anxiolytic neurotransmitter GABA, is supposed to influence various mental disorders, including mood and anxiety disorders. In a recent association study in depression, which is highly comorbid with panic disorder, *GAD1* risk allele associations were restricted to females.

**Methodology/Principal Findings:**

Nineteen single nucleotide polymorphisms (SNPs) tagging the common variation in *GAD1* were genotyped in two independent gender and age matched case-control samples (discovery sample n = 478; replication sample n = 584). Thirteen SNPs passed quality control and were examined for gender-specific enrichment of risk alleles associated with panic disorder by using logistic regression including a genotype×gender interaction term. The latter was found to be nominally significant for four SNPs (rs1978340, rs3762555, rs3749034, rs2241165) in the discovery sample; of note, the respective minor/risk alleles were associated with panic disorder only in females. These findings were not confirmed in the replication sample; however, the genotype×gender interaction of rs3749034 remained significant in the combined sample. Furthermore, this polymorphism showed a nominally significant association with the Agoraphobic Cognitions Questionnaire sum score.

**Conclusions/Significance:**

The present study represents the first systematic evaluation of gender-specific enrichment of risk alleles of the common SNP variation in the panic disorder candidate gene *GAD1*. Our tentative results provide a possible explanation for the higher susceptibility of females to panic disorder.

## Introduction

The lifetime prevalence of anxiety disorders has been estimated to be as high as 30% with a spectrum that ranges from 2% for agoraphobia up to 12% for specific phobia; with the prevalence of panic disorder being estimated at 5% [1]. Of note, the prevalence of panic disorder is twice as high in females as in males [2]. Panic disorder displays a high comorbidity with other anxiety (e.g. agoraphobia) and mood disorders (e.g. unipolar depression), which phenotypically share the personality trait neuroticism [3], [4]. Current drug treatment involves the use of benzodiazepines, whose anxiolytic effect is caused by increasing the responsiveness of the γ-aminobutyric acid (GABA) receptor [5]. Correspondingly, the results of several magnetic resonance spectroscopy studies point to an impaired GABA system in panic disorder patients [6], [7]. The key enzyme for the synthesis of the inhibitory neurotransmitter GABA is glutamic acid dehydrogenase (GAD) which uses glutamate and pyridoxal phosphate as substrates for the reaction that takes place in presynaptic neurons (reviewed in [8]); it exists in two isoforms – 67 kDa and 65 kDa – encoded by different genes (*GAD1*, located at 2q31.1 and *GAD2* (10p11.23), respectively). Intriguingly, brain *GAD* expression was shown to be influenced by sex hormones in female rats and rhesus macaques [9], [10]. In the latter species, estrogen and progesterone decrease *GAD* expression in the amygdala and the hippocampus (which both are involved in regulating fear), which provides a link between hormone levels and anxiety as well as mood attacks during menstruation in humans [10].

A recent twin study estimated the heritability of panic disorder at 48% [3] and an independently conducted genome-wide linkage scan yielded susceptibility loci at chromosomal regions 2q (including *GAD1*) and 15q [11]. With the assumption that both *GAD* genes may serve as plausible candidates to influence neuroticism, the examination of allelic variation in these genes revealed associations of *GAD1* single nucleotide polymorphisms (SNPs) rs2241165, rs2058725 and rs3791850 in a mixed anxiety (including panic) and mood disorder sample [12]. Studies in bipolar disorder further corroborate the association of *GAD1* with mood disorders (rs1978340, rs872123 and rs2241165) [13], [14], but also in schizophrenia, *GAD1* alleles were shown to convey genetic risk (rs10432420, rs3749035, rs16823181, rs3791878, rs3791858, rs3749034, rs2270335, rs2241165, rs379850) [15], [16], [17], [18]. Recently, the *GAD1* SNPs examined in [12] were (along with SNPs mapping to other genes) explored in an independent sample of patients suffering from major depression, with a particular focus on sleep disturbance subtypes and gender [19]. Those *GAD1* SNPs that were not (rs12185692) or only marginally (rs769407) associated with neuroticism [12] revealed female-specific associations with depression [19]. Since depression as well as panic disorder are both more prevalent among females, this striking gender difference prompted us to investigate whether gender-specific associations of *GAD1* variants are also detectable in panic disorder. In the present study we used two independent gender-matched case/control samples with a total size of n_case_ = 531 to test this hypothesis. Nominally significant gender differences and associations with disease were found in the discovery sample, which were however not supported by the replication sample; in the analysis of the combined sample, only one SNP displayed a nominally significant gender-specific risk allele enrichment, the reasons for which are discussed in depth below. Studies in larger samples and subsequent meta-analysis are thus needed to unequivocally prove a possible female-specific contribution of the *GAD1* gene in panic disorder.

## Results

In order to capture the common allelic variation in *GAD1* and approximately 10 kb of the gene's up- and downstream flanking regions, we chose a representative set of 19 SNPs (based on HapMap CEU data) for genotyping. Thereof, six SNPs did not pass our stringent quality criteria in the discovery sample; hence, the remaining set of 13 SNPs (see Table S1) was still found to be representative for >80% of common HapMap SNPs in the defined region. In a first analysis step, we tested our *a priori* hypothesis by modeling panic disorder disease risk with the number of risk alleles conditioning on gender, implementing a multiple regression including genotype×gender interactions in statistical terms. The odds ratio (OR) derived from this interaction term represents the ratio of effect sizes (i.e. ORs) from genetic associations in males versus those in females, thus quantifying differential risk allele enrichment between genders. In the discovery sample, four SNPs displayed nominally significant genotype×gender interactions; particularly the respective minor alleles (assumed to convey the genetic risk) of rs3762555, rs3749034 and rs2241165 had effects that were almost twice as large in males as in females (ORs close to 2) and *vice versa* for rs1978340 (OR close to 0.5; see Table 1). These four candidate SNPs were subjected to a *post hoc* analysis in which we tested the genotype associations with panic disorder separately in each gender; this aimed at investigating whether these nominally significant gender interactions go along with SNP associations in both genders, however with opposing effects. Indeed, the estimated effect sizes for the four SNPs were opposite between genders (i.e. risk/OR>1 versus protective/OR<1), however, associations reached nominal significance only in females (see Table 2). Notably, the associations of rs3762555, rs3749034 and rs2241165 are unlikely to be independent because these SNPs are located on the same haplotype block (see Figure S1).

**Table 1:** Genotype×gender interaction analysis of common *GAD1* alleles associated with panic disorder.

*SNP*	*Alleles*	*Discovery*		*Replication*		*Combined*	
*rs ID*	*minor/major*	*OR*	*p*	*OR*	*p*	*OR*	*p*
**rs1978340**	**A/G**	**0.519**	**0.032**	1.292	0.406	0.832	0.390
rs3791878	T/G	0.578	0.067	1.443	0.241	0.938	0.764
**rs3762555**	**C/G**	**1.872**	**0.041**	1.446	0.279	1.496	0.068
**rs3749034**	**A/G**	**1.992**	**0.023**	1.445	0.291	**2.009**	**0.045**
rs2270335	T/C	1.713	0.073	4.060	0.429	1.398	0.121
**rs2241165**	**C/T**	**1.958**	**0.030**	1.325	0.411	1.483	0.076
rs11542313	C/T	0.979	0.936	0.691	0.226	0.826	0.332
rs3828275	T/C	0.780	0.365	0.607	0.101	0.718	0.096
rs2058725	C/T	1.763	0.058	1.296	0.438	1.472	0.074
rs701492	T/C	0.745	0.338	0.909	0.760	0.828	0.380
rs16858996	G/A	0.624	0.294	1.565	0.412	0.965	0.914
rs17701824	T/C	1.072	0.795	1.067	0.823	1.032	0.870
rs4439928*	G/A	0.435	0.098	---	---	---	---

* rs4439928 did not meet the quality criteria in the replication sample.

Nominally significant results are shown in bold. Abbreviations: OR, odds ratio; p, p-value; SNP, single nucleotide polymorphism.

**Table 2:** Gender-wise association of *GAD1* polymorphisms displaying gender-specific risk allele enrichment with panic disorder.

*SNP*	*Alleles*	*Gender*	*Discovery*		*Replication*		*Combined*	
*rs ID*	*minor/major*		*OR*	*p*	*OR*	*p*	*OR*	*p*
rs1978340	A/G	**female**	**1.588**	**0.012**	1.004	0.978	1.221	0.091
		male	0.825	0.429	1.298	0.326	1.016	0.929
rs3762555	C/G	**female**	**0.648**	**0.030**	1.082	0.614	0.888	0.330
		male	1.213	0.407	1.565	0.139	1.328	0.122
rs3749034	A/G	**female**	**0.623**	**0.019**	4.066	0.753	0.862	0.222
		male	1.242	0.336	1.518	0.181	1.335	0.110
rs2241165	C/T	**female**	**0.615**	**0.016**	1.106	0.520	0.881	0.299
		male	1.205	0.427	1.465	0.210	1.306	0.15

Nominally significant results are shown in bold. Abbreviations: OR, odds ratio; p, p-value; SNP, single nucleotide polymorphism.

In order to replicate these initial findings, the 13 SNPs examined in the discovery sample were genotyped in an independent replication sample; thereof, genotyping results of rs4439928 did not meet the quality criteria and accordingly this SNP was excluded from replication analysis. The overall linkage disequilibrium structure of the replication sample was found to be similar to the discovery sample (see Figure S2). However, in contrast to the results from the discovery sample, none of the gender-specific risk allele enrichments reached even marginal (p<0.1) significance (see Table 1). Furthermore, none of the four candidate SNPs showed the trend to be associated with panic disorder in gender-specific subsets of the replication sample (see Table 2). Despite this apparently failed replication, it should be noted that the number of male probands (n = 152) as well as the male proportion of the replication sample (26%) are smaller than those of the discovery sample (n = 192, 40%). In order to exclude false positive as well as false negative associations as a result from insufficient sampling, we decided to further analyze the combined sample. In this setting, three of four significant gender differences found in the discovery sample gained at least marginal significance (see Table 1). However, the four candidate SNPs were not associated with panic disorder in gender subsets (see Table 2).

Since the replication sample offers the advantage of known dimensional phenotypes, we furthermore aimed to quantify the effect of our candidate SNPs on ASI and ACQ sum scores. The range of these scores differs between healthy individuals and panic patients per definition, resulting in unequal variances between groups. We therefore determined gender-wise genotype associations with either sum score separately for the panic and the control groups. In all but one case, the estimated effects conveyed by each risk allele were smaller in males compared to females, however, only two estimated effects were significantly different from zero (see Table 3). Although largely insignificant, these results lend qualitative support to our hypothesis that *GAD1* alleles may have a different impact on panic disorder susceptibility in both genders.

**Table 3:** Associations of *GAD1* candidate SNPs with dimensional anxiety traits.

*Score*	*SNP*	*Alleles*	*Gender*	*Panic*		*Control*	
	*rs ID*	*minor/major*		*increase*	*p*	*increase*	*p*
ACQ	rs1978340	A/G	female	0.043	0.512	0.008	0.782
			male	−0.019	0.848	−0.040	0.260
	rs3762555	C/G	female	−0.024	0.728	0.018	0.519
			male	−0.196	0.068	−0.007	0.873
	**rs3749034**	**A/G**	female	−0.028	0.683	0.026	0.351
			**male**	**−0.263**	**0.027**	−0.007	0.868
	rs2241165	C/T	female	−0.006	0.932	0.014	0.615
			male	−0.201	0.080	−0.002	0.971
ASI	**rs1978340**	**A/G**	female	1.891	0.130	0.094	0.902
			**male**	−0.837	0.714	**−2.641**	**0.015**
	rs3762555	C/G	female	−1.051	0.423	0.462	0.524
			male	−1.562	0.528	−0.154	0.907
	rs3749034	A/G	female	−1.328	0.312	0.650	0.361
			male	−1.569	0.552	−0.218	0.868
	rs2241165	C/T	female	−1.153	0.394	0.595	0.398
			male	−0.883	0.728	−0.441	0.733

Nominally significant results are shown in bold. Abbreviations: ACQ, Agoraphobic Cognitions Questionnaire; ASI, Anxiety Sensitivity Index; OR, odds ratio; p, p-value; SNP, single nucleotide polymorphism.

In addition to genetic association testing we tried to predict functional consequences of our candidate SNPs using a bioinformatic approach. We found that SNPs close to *GAD1's* transcription start site are linked to putative changes of promoter function. Particularly, the minor allele of rs1978340 (upstream) deletes a putative transcription factor binding site (TFBS) for the E2F4-TFDP2 dimer, whereas the minor alleles of rs3762555 (upstream) and rs3749034 (5′ untranslated region) create predicted TFBSs for SRF and ZEB1/ZFHEP/AREB6, respectively. For the intronic SNP rs2241165 no allele-specific functional consequences (affecting e.g. splicing) were evident, nor is linkage disequilibrium data available for nearby SNPs with putative structural impact mediated by amino acid exchanges (rs77655188/*GAD1^T27K^*; rs17857148/*GAD1^H291R^*; rs17857149/*GAD1^K318E^*). The restriction of this analysis to our candidate SNPs imposes the limit that true causal variants may be missed, since each of our SNPs examined in this study is representative for the variation of several polymorphisms. Nevertheless, it appears conceivable that the mode of action of gender-wise differentially enriched *GAD1* risk alleles affects promoter function and thus transcriptional efficiency.

## Discussion

In this paper, we present the first systematic evaluation of gender-dependent associations of common SNP variation in *GAD1* with panic disorder. However, the initial findings from our clinical discovery sample were not supported by our replication sample originating from a cognitive behavioral psychotherapy (CBT) trial. Of four genotype associations, no replicated association was found at the categorical level and in the combined sample only the interaction of rs3749034 with gender became significant. Interestingly though, rs3749034 was also among the SNPs associated with the dimensional sum score of ACQ in the replication sample, but at first sight this dimensional association seems to contradict the categorical results: although the latter revealed significant gender-differences, rs3749034 had a protective effect on all females in the discovery sample and on male panic patients in the replication sample as well. However, since the ACQ quantifies agoraphobia [20], the analyses of our dimensional and categorical phenotypes need not necessarily reveal concordant results. This point is of particular interest because the discovery and replication samples differ in their comorbidity with agoraphobia (see below and Table S2). Finally, a concordantly significant reduction of ACQ in male replication controls was also not detectable, arguing for the possibility that the association of rs3749034 with ACQ in male panic patients may be a false positive.

A major limitation of this study is imposed by the restricted sample size and the high female to male ratio. Due to the high LD within the examined region, we used nominal p-values for the assessment of significance to avoid the (in this case) overly strict Bonferroni correction, which revealed associations in the discovery sample that would otherwise have been negative. This measure decreases type-II error, while increasing type-I error, which has to be taken into account when interpreting the data. Due to the lower prevalence of panic disorder in males, especially the male subsamples were rather limited in size, which may hinder the realistic estimation of genetic effect sizes because of insufficient sampling and therefore confound possible gender differences in risk allele enrichment. This point is more relevant to the replication sample, which features an even smaller male sub-sample as compared to the discovery sample, and which did not support the findings from the discovery sample. As the combined sample did not reflect our associations from the discovery sample either, it is tempting to speculate that lack of replication may also be explained by differences in the composition of our samples (see Table S2): Firstly, mean age of discovery cases (37.59±11.13) and controls (36.18±11.78) were more similar in the discovery than in the replication sample (cases, 36.04±10.77 vs. controls, 28.8±7.38). Due to the younger age of replication controls, some of the apparently healthy controls at the time of blood donation may not have reached the age of onset for panic disorder, so that the fraction of persons predisposed to panic disorder may be higher in replication than in discovery controls [1]. On the other hand, replication controls were screened against mental axis 1 disorders, whereas discovery controls were anonymous blood donors. Nevertheless the likelihood of severe psychiatric disorders present in the control sample is assumed to be low, because blood donors were informed about the intended enrollment in this study. Therefore, given the panic disorder prevalence of 5% [1], in a worst-case scenario the replication controls (n = 292) may contain up to 15 panic disorder patients, which should result in only a minor distortion of the OR. Secondly, the patients of the discovery sample were a mixed in- and outpatient sample from psychiatric departments recruited with the primary diagnosis of panic disorder, while the patients of the replication sample were all outpatients from psychological outpatient services volunteering for a CBT trial of panic with agoraphobia and thus recruited with the primary diagnosis panic disorder with agoraphobia. Of the 239 panic patients of our discovery sample, only 68.6% (n = 164) thus were diagnosed with comorbid agoraphobia, whereas this was the case for all 292 panic patients of the replication sample. Despite the high degree of comorbidity of panic disorder and agoraphobia, the genetic architecture may differ between the two samples. This notion is supported by results from association tests with swapped samples: discovery cases versus replication cases again revealed significant differences in some of the associated markers of the discovery sample; discovery controls and replication controls however were not different. On the other hand, discovery cases versus replication controls partly confirmed the discovery findings, but not replication cases versus discovery controls (see Table S3). Thus, the observed gender differences may be caused by “pure” panic patients in the discovery sample (n = 75). Further exploratory analyses however revealed a trend for gender-specific associations in sub-samples of panic disorder with and without comorbid agoraphobia (see Table S4).

In conjunction with the present genetic data, convergent lines of evidence suggest that *GAD1* and its gene product are implicated in the pathophysiology of panic disorder. GABA levels in various brain regions are reduced in panic patients [6], [7], possibly due to impaired GAD function. Further studies in patients with major depression, a mechanistically related disorder, found reduced GABA levels to be accompanied by increased glutamate concentrations [21], strengthening the link between anxiety and mood disorders and GAD. In patients with stiff person syndrome (SPS) GAD function is decreased due to the development of auto-antibodies against this enzyme; these are not only responsible for the primary phenotype – increased muscle stiffness and intermittent muscle spasms – but also raise anxiety in SPS patients [22], [23]. This connection is particularly supported by the fact that passive transfer of GAD antibodies to healthy rats resulted in an anxious phenotype in these animals was well [24]. These observations are complemented with genetic evidence, which shows that *GAD1* SNPs are associated with neuroticism (rs2241165, rs2058725 and rs3791850) and anxiety (rs769407, rs3791851 and rs769395) [12], [25]. Furthermore, the *GAD1* polymorphism rs1978340 was found to influence brain GABA levels: healthy individuals that are homozygous for the minor allele display higher concentrations than major allele carriers [26]. Intriguingly, this seems to contradict our result that rs1978340 is associated with increased risk for panic disorder in females (see Table 2). However, this particular SNP's effect on the risk to be affected by schizophrenia, a disorder that also features reduced brain GABA levels [27], seems to depend on the *COMT* promoter polymorphism rs2075507 [17], so that epistasis seems to play an important role. This however remains elusive with respect to GABA concentrations.

Further genetic studies on psychiatric disorders are in line with our tentative finding of gender-specific enrichment of *GAD1* risk alleles in panic disorder. In the latter, a linkage analysis of an US American sample revealed that both chromosome 2 microsatellite marker loci, which flank the *GAD1* gene at positions 169 and 178 cM, display moderate differences in male and female associations [11]. In unipolar depression, which is comorbid with panic disorder, a Finnish association study found alleles of the *GAD1* polymorphisms rs12185692 (upstream) and rs769407 (in the sixth intron) to go along with disease only in females [19]. Furthermore, several studies on schizophrenia also revealed gender differences in associations of *GAD1* SNPs [15], [16], [17]. An overview of gender-specific SNP associations is given in Table S5. In line with this, other association studies examining *GAD1* in mixed gender samples of depression [28], schizophrenia [29] and autism [30], [31] revealed no associations. This also holds true for the examination of our samples without subgrouping by gender (see Table S6). On the other hand, the studies on neuroticism and anxiety demonstrated *GAD1* SNP associations even without considering gender effects [12], [25]. Compared to our discovery sample, the latter studies employed a larger sample size, which increases the power to detect smaller genetic effects.

Since the heritability for panic disorder is somewhat lower than that for other mental disorders [32], [33], non-genetic (i.e. environmental) influences contribute to a large part to the etiology of panic disorder. One important class of environmental influences in terms of panic disorder are life events [34]. Recently, in an experimental paradigm it was shown that low maternal care – which might serve as an animal experimental model for early life stress in humans – leads to increased CpG methylation as well as to decreased H3K9 acetylation of the *Gad1* promoter in the rat hippocampus, thus silencing gene expression [35]. It is therefore conceivable that life events and underlying molecular mechanisms like e.g. DNA methylation and histone acetylation may interfere with genetic effects. Such a gene×environment correlation of *GAD1* SNPs and childhood adversities has very recently been examined in the context of anxiety disorders, however failed to detect significant effects [25]. In the light of hormone-induced gender-dependent *GAD1* expression [10] however, a gene×environment×gender correlation seems plausible which has not been tested for in this study.

In summary, our study provides supporting evidence for gender differences in the role of *GAD1* variation for the pathogenesis of panic disorder, but is far from conclusive. Future studies in larger gender-balanced samples well characterized for panic disorder and agoraphobia as well as comorbid psychopathology (including dimensional phenotypes) and life events will be necessary to evaluate the relevance of our tentative findings.

## Materials and Methods

### Sample

All SNPs examined in this study were genotyped in panic disorder (with or without agoraphobia) patients which were matched by gender to an equal number of healthy controls. Details on case/control pairs of the discovery and the replication sample are specified below. All cases as well as controls were unrelated and of self-reported Western European descent. Patients with comorbid schizoaffective or other psychotic disorders, substance abuse disorders, mental retardation, neurological or neurodegenerative disorders were excluded. Only patients and volunteers who gave written informed consent were enrolled in the study. The present study complied with the Declaration of Helsinki and was specifically approved by the Ethics Committee of the University Hospital, University of Würzburg. A demographic overview of the discovery and the replication samples can be found in Table S2.

The discovery sample consisted of 239 panic disorder patients, collected in Bonn, Würzburg, Münster and Göttingen (female = 143, male = 96; mean age 37.59±11.13), from out- and inpatients of the respective centers treated there as part of the regular clinical care. The diagnosis of panic disorder and absence or presence of comorbid agoraphobia (68.6%; n = 164) was ascertained by experienced clinicians on the basis of medical records and structured or standardized clinical interviews (Schedule for Affective Disorders and Schizophrenia (lifetime version), SADS-LA; Structured Clinical Interview for DSM IV, SCID; and Composite Inernational Diagnostic Interview, CIDI) according to the criteria of DSM- (Diagnostic and Statistical Manual of Mental Disorders) III-R or DSM-IV, respectively [36], [37], [38]. The gender-matched control sample comprised 239 anonymous blood donors of German descent (female = 143, male = 96; mean age 36.18±11.78) that were not screened for psychiatric disorders. However, all apparently healthy individuals were aware of their intended enrollment in the control sample of the study and furthermore, as a requirement for blood donation, were free of medication. Therefore the likelihood of severe psychiatric disorders present in the control sample was assumed to be low.

The replication sample's patients (n = 292; female = 216, male = 76; mean age 36.04±10.77) were enrolled from the BMBF “*Panic-Net*” multicenter psychotherapy treatment study; 15 of these patients did not enter the study yet fulfilled the respective inclusion criteria. Patients were enrolled from specialized outpatient treatment units within the framework of the Panic-Net study as described in detail [39]. The diagnosis of panic disorder with agoraphobia was established by a standardized clinical interview (CIDI) according to DSM-IV criteria [38]. Controls used for genotypic associations with panic disorder (n = 292; female = 216, male = 76; mean age 28.8±7.38) were from Münster and Würzburg, matched by gender to cases and drawn from a larger number of screened healthy controls (n = 1564; female = 809, male = 755; mean age 24.93±5.11 ascertained within the framework of the Collaborative Research Center TRR SFB 58). Absence of mental axis 1 disorders was established by experienced psychologists on the basis of a structured clinical interview (Mini International Neuropsychiatric Interview, MINI) according to the criteria of DSM-IV [38]. For both patient and control groups, panic fear and anxiety sensitivity were evaluated by German versions of the Anxiety Sensitivity Index (ASI, [40]) and Agoraphobic Cognitions Questionnaire (ACQ, [20]).

### Genotyping

In order to capture allelic variation in the *GAD1* gene, 19 tag SNPs were derived from HapMap CEU data [41] using the *Tagger* function as implemented in Haploview V4.2 [42] with default settings. SNP genotyping was performed using Sequenom's MassArray® system according to the instructions supplied by the manufacturer. All PCR reactions were done with the iPlex® chemistry following the MassArray® iPlex® standard operation procedure. Primer sequences can be found in Table S7.

### Statistical Analysis

Prior to statistical analysis, SNPs had to fulfill several quality control criteria. The minimal call rate threshold was set to 90%, SNPs with a call rate below this threshold (rs769390, rs4668331 and rs4439928, the latter only in the replication sample) where not further analyzed. Deviations from Hardy-Weinberg equilibrium (HWE) were considered to be indicative for the presence of genotyping errors; SNPs rs769393 and rs769395 yielded a HWE p-value below the threshold of 0.01 and were thus excluded from further analysis. Also, rare variants with a minor allele frequency (MAF) below 5% (rs769406, rs12472230) were not further examined. The resulting set of 13 SNPs tagging the common allelic variation in the *GAD1* gene was analyzed with logistic regression, assuming the minor alleles to convey the genetic risk in an additive manner. SNP associations with dimensional anxiety traits were analyzed with univariate linear regressions modeling the ACQ and the ASI sum scores in each gender separately. Each risk allele's effect is presented as OR for categorical outcomes or as linear increase for dimensional phenotypes. The respective p-values represent the probabilities for the estimated effects being indistinguishable from zero. P-values <0.05 are termed nominally significant. Although conducting repeated statistical tests, p-values were not corrected for multiple testing, which was – despite large sample size - due to the limited power of the study. Given a SNP with MAF = 5% conveying a moderate relative risk of 1.5 to develop panic disorder in an additive model, the power to find a nominal association (p<0.05) is 65% for the mixed gender combined sample (n = 1062), 48% for its female (n = 718) and 26% for its male (n = 344) subset. In the same analysis setting, but split up in discovery and replication sample, the mixed gender discovery sample (n = 478) and its female (n = 286) and male (n = 192) subsets provide 35%, 24% and 18% power, respectively; the mixed gender replication sample (n = 584) and its female (n = 432) and male (n = 152) subsets provide 42%, 32% and 15% power, respectively. The analyses were performed with Haploview V4.2 [42], PGA [43], PLINK V1.07 [44] and R V2.11 [45].

### Assessment of SNP function

Analyses of SNPs and their sequence contexts were performed with tools that are contained in the GenEpi toolbox [46]. Differential TFBS predictions were made using the web-based tool MatInspector [47]. Summary tables produced by F-SNP [48], [49] were used to indicate a SNP's possible influence on splicing junctions.

## Supporting Information

Figure S1 **Linkage disequilibrium (LD) structure of** ***GAD1*** **single nucleotide polymorphisms examined in the discovery sample.** LD analysis was performed with Haploview v4.2 using default settings, i.e. D′ was used as measure for LD and haplotype blocks were defined with the method “confidence intervals”.
(DOC)

Figure S2 **Linkage disequilibrium (LD) structure of** ***GAD1*** **single nucleotide polymorphisms examined in the replication sample.** LD analysis was performed with Haploview v4.2 using default settings, i.e. D′ was used as measure for LD and haplotype blocks were defined with the method “confidence intervals”.
(DOC)

Table S1 **Single nucleotide polymorphisms (SNPs) examined in the study.** rs4439928 did not meet the quality criteria in the replication sample and was therefore not included in the replication and combined sample analysis.
(DOC)

Table S2 **Demographic overview of the discovery and the replication sample.** Abbreviations: AG, agoraphobia; CIDI, Composite International Diagnostic Interview; DSM, Diagnostic and Statistical Manual of Mental Disorders; MINI, Mini International Neuropsychiatric Interview; SADS-LA, Schedule for Affective Disorders and Schizophrenia (lifetime version); SCID, Structured Clinical Interview for DSM IV.
(DOC)

Table S3 **Gender-specific associations of** ***GAD1*** **polymorphisms in swapped samples.** Analysis of gender subsamples was performed only for polymorphisms that displayed significant gender differences in the discovery case-control sample (see Table 1); rs4439928 did not meet the quality criteria in the replication sample and was therefore not included in the swapped sample analysis. Nominally significant results are shown in bold. Abbreviations: Ca, case group; Co, control group; D, discovery sample; OR, odds ratio; p, p-value; R, replication sample; SNP, single nucleotide polymorphism.
(DOC)

Table S4 **Gender-specific associations of** ***GAD1*** **polymorphisms in samples with and without co-morbid agoraphobia.** Analysis of gender subsamples was performed only for polymorphisms that displayed significant gender differences in the discovery case-control sample (see Table 1); rs4439928 did not meet the quality criteria in the replication sample and was therefore not included in the analysis of the combined sample. Nominally significant results are shown in bold. Abbreviations: AG, agoraphobia; D, discovery sample; OR, odds ratio; p, p-value; R, replication sample; SNP, single nucleotide polymorphism.
(DOC)

Table S5 **Published gender-specific associations of** ***GAD1*** **single nucleotide polymorphisms (SNP).**
(DOC)

Table S6 **Associations of** ***GAD1*** **polymorphisms with panic disorder in mixed-gender samples.** rs4439928 did not meet the quality criteria in the replication sample and was therefore not included in the replication and combined mixed-gender sample analysis. Abbreviations: OR, odds ratio; p, p-value; SNP, single nucleotide polymorphism.
(DOC)

Table S7 **Primer sequences used in this study.**
(DOC)
