# Supplementary figures and images for: Gender Differences in Associations of Glutamate Decarboxylase 1 Gene (GAD1) Variants with Panic Disorder
Source: PLoS One. 2012 May 25;7(5):e37651. doi: 10.1371/journal.pone.0037651 (PMC3360757; doi:10.1371/journal.pone.0037651)

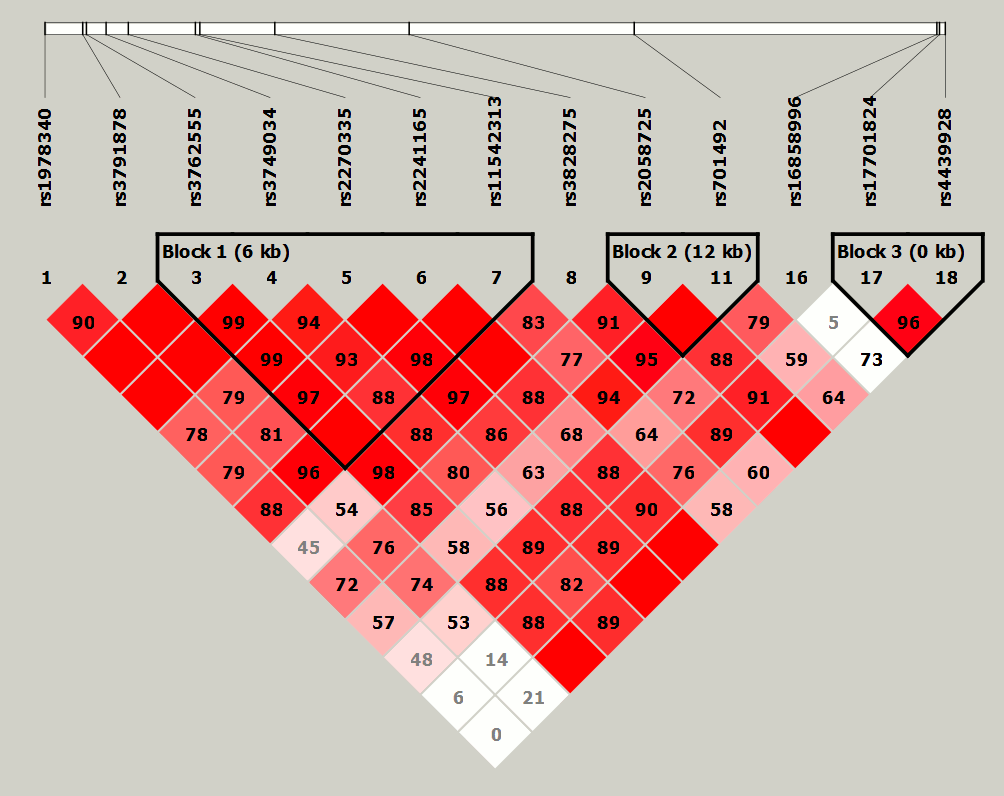

Supplement: Figure S1 — Linkage disequilibrium (LD) structure of GAD1 single nucleotide polymorphisms examined in the discovery sample. LD analysis was performed with Haploview v4.2 using default settings, i.e. D′ was used as measure for LD and haplotype blocks were defined with the method “confidence intervals”. (DOC) [file pone.0037651.s001.doc]

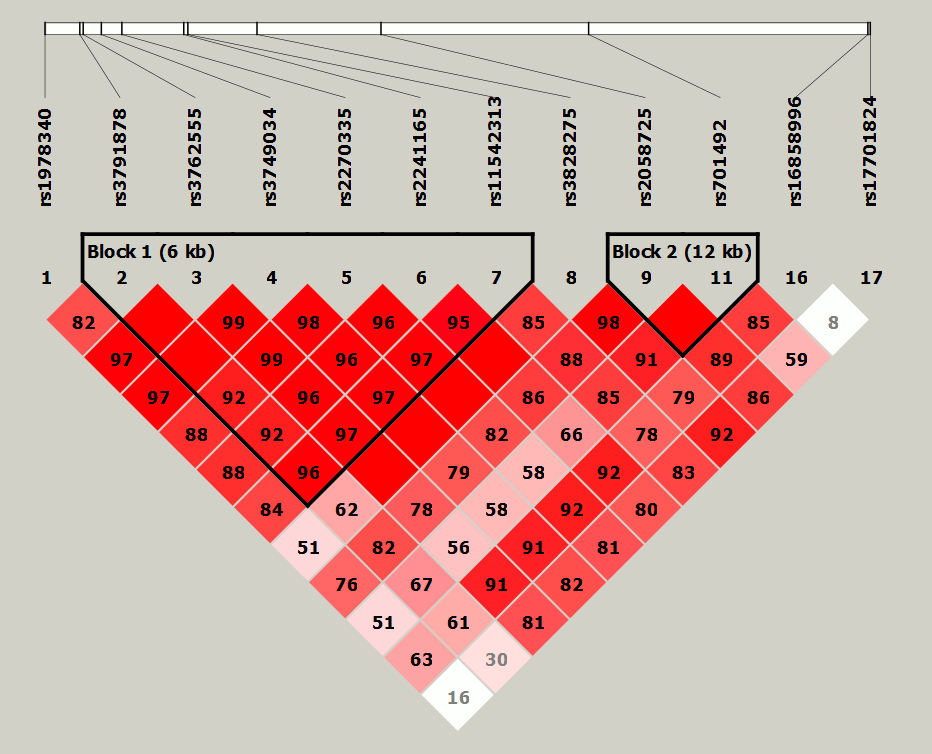

Supplement: Figure S2 — Linkage disequilibrium (LD) structure of GAD1 single nucleotide polymorphisms examined in the replication sample. LD analysis was performed with Haploview v4.2 using default settings, i.e. D′ was used as measure for LD and haplotype blocks were defined with the method “confidence intervals”. (DOC) [file pone.0037651.s002.doc]
