# Supplementary material for: Gender Differences in Associations of Glutamate Decarboxylase 1 Gene (GAD1) Variants with Panic Disorder
Source: PLoS One. 2012 May 25;7(5):e37651. doi: 10.1371/journal.pone.0037651 (PMC3360757; doi:10.1371/journal.pone.0037651)
Supplement: Table S1 — Single nucleotide polymorphisms (SNPs) examined in the study. rs4439928 did not meet the quality criteria in the replication sample and was therefore not included in the replication and combined sample analysis. (DOC) [file pone.0037651.s003.doc]

| ***SNP*** | ***Position*** | ***Alleles*** | ***Minor Allele Frequency*** | | |
| --- | --- | --- | --- | --- | --- |
| ***rs ID*** | ***basepair*** | ***minor / major*** | ***Discovery*** | ***Replication*** | ***Combined*** |
| rs1978340 | 171670121 | A/G | 0.280 | 0.294 | 0.287 |
| rs3791878 | 171672191 | T/G | 0.298 | 0.310 | 0.304 |
| rs3762555 | 171672395 | C/G | 0.260 | 0.250 | 0.255 |
| rs3749034 | 171673475 | A/G | 0.267 | 0.244 | 0.254 |
| rs2270335 | 171674696 | T/C | 0.271 | 0.256 | 0.263 |
| rs2241165 | 171678379 | C/T | 0.267 | 0.261 | 0.264 |
| rs11542313 | 171678625 | C/T | 0.420 | 0.409 | 0.414 |
| rs3828275 | 171682740 | T/C | 0.439 | 0.421 | 0.429 |
| rs2058725 | 171690121 | C/T | 0.277 | 0.250 | 0.262 |
| rs701492 | 171702480 | T/C | 0.249 | 0.288 | 0.271 |
| rs16858996 | 171719102 | G/A | 0.093 | 0.089 | 0.091 |
| rs17701824 | 171719246 | T/C | 0.463 | 0.474 | 0.469 |
| rs4439928 | 171719565 | G/A | 0.102 | -- | -- |
