# Supplementary material for: Gender Differences in Associations of Glutamate Decarboxylase 1 Gene (GAD1) Variants with Panic Disorder
Source: PLoS One. 2012 May 25;7(5):e37651. doi: 10.1371/journal.pone.0037651 (PMC3360757; doi:10.1371/journal.pone.0037651)
Supplement: Table S2 — Demographic overview of the discovery and the replication sample. Abbreviations: AG, agoraphobia; CIDI, Composite International Diagnostic Interview; DSM, Diagnostic and Statistical Manual of Mental Disorders; MINI, Mini International Neuropsychiatric Interview; SADS-LA, Schedule for Affective Disorders and Schizophrenia (lifetime version); SCID, Structured Clinical Interview for DSM IV. (DOC) [file pone.0037651.s004.doc]

|  | **Discovery sample** | | **Replication sample** | |
| --- | --- | --- | --- | --- |
|  | **Cases** | **Controls** | **Cases** | **Controls** |
| Total (+AG/-AG)  Females (+AG/-AG)  Males (+AG/-AG) | N=239 (164/75)  N=143(108/35)  N=96 (56/40) | N=239  N=143  N=96 | N=292 (292/0)  N=216 (216/0)  N=76 (76/0) | N=292  N=216  N=76 |
| Age | 37.59+11.13 | 36.18+11.78 | 36.04±10.77 | 28.8±7.38 |
| Descent | Western European | German | Western European | Western European |
| Recruitment site | Bonn, Goettingen, Muenster, Wuerzburg | Muenster | Aachen, Berlin, Dresden, Greifswald Muenster, Wuerzburg, | Muenster, Wuerzburg |
| Diagnostic instruments | SADS-LA, DSM-IV, SCID, CIDI | -- | CIDI, DSM-IV | MINI, DSM-IV |
| Inclusion criteria | DSM-III-R or DSM-IV | -- | DSM-IV | absence of mental axis 1 disorders |
