# Supplementary material for: Gender Differences in Associations of Glutamate Decarboxylase 1 Gene (GAD1) Variants with Panic Disorder
Source: PLoS One. 2012 May 25;7(5):e37651. doi: 10.1371/journal.pone.0037651 (PMC3360757; doi:10.1371/journal.pone.0037651)
Supplement: Table S3 — Gender-specific associations of GAD1 polymorphisms in swapped samples. Analysis of gender subsamples was performed only for polymorphisms that displayed significant gender differences in the discovery case-control sample (see Table 1); rs4439928 did not meet the quality criteria in the replication sample and was therefore not included in the swapped sample analysis. Nominally significant results are shown in bold. Abbreviations: Ca, case group; Co, control group; D, discovery sample; OR, odds ratio; p, p-value; R, replication sample; SNP, single nucleotide polymorphism. (DOC) [file pone.0037651.s005.doc]

| ***SNP*** | ***Coefficient*** | ***RCa vs DCa*** | | ***DCo vs RCo*** | | ***DCa vs RCo*** | | ***RCa vs DCo*** | |
| --- | --- | --- | --- | --- | --- | --- | --- | --- | --- |
| ***rs ID*** |  | ***OR*** | ***p*** | ***OR*** | ***p*** | ***OR*** | ***p*** | ***OR*** | ***p*** |
| rs1978340 | interaction | 1.841 | 0.051 | 1.423 | 0.246 | 0.722 | 0.288 | 0.916 | 0.776 |
| rs1978340 | female | 0.785 | 0.147 | 0.781 | 0.160 | 1.285 | 0.139 | 1.280 | 0.155 |
| rs1978340 | male | 1.445 | 0.163 | 0.900 | 0.672 | 0.928 | 0.770 | 1.173 | 0.531 |
| rs3791878 | interaction | 1.638 | 0.109 | 1.519 | 0.166 | 0.852 | 0.587 | 0.983 | 0.956 |
| rs3762555 | interaction | 0.524 | **0.044** | 1.384 | 0.313 | 2.505 | **0.005** | 1.007 | 0.983 |
| rs3762555 | female | 1.456 | **0.045** | 1.151 | 0.411 | 0.769 | 0.140 | 0.932 | 0.696 |
| rs3762555 | male | 0.763 | 0.299 | 0.628 | 0.088 | 1.927 | **0.016** | 0.939 | 0.810 |
| rs3749034 | interaction | 0.473 | **0.021** | 1.437 | 0.254 | 2.735 | **0.002** | 0.961 | 0.901 |
| rs3749034 | female | 1.430 | 0.060 | 1.187 | 0.314 | 0.751 | 0.116 | 0.876 | 0.456 |
| rs3749034 | male | 0.675 | 0.135 | 0.586 | **0.047** | 2.055 | **0.006** | 0.841 | 0.522 |
| rs2270335 | interaction | 0.547 | 0.057 | 1.326 | 0.368 | 2.254 | **0.011** | 0.974 | 0.932 |
| rs2241165 | interaction | 0.516 | **0.048** | 1.247 | 0.480 | 2.409 | **0.007** | 1.041 | 0.900 |
| rs2241165 | female | 1.565 | **0.021** | 1.162 | 0.367 | 0.734 | 0.089 | 0.944 | 0.744 |
| rs2241165 | male | 0.808 | 0.432 | 0.690 | 0.161 | 1.768 | **0.034** | 0.983 | 0.949 |
| rs11542313 | interaction | 1.152 | 0.622 | 0.637 | 0.104 | 0.626 | 0.093 | 1.119 | 0.695 |
| rs3828275 | interaction | 1.213 | 0.516 | 0.705 | 0.192 | 0.510 | **0.022** | 0.944 | 0.835 |
| rs2058725 | interaction | 0.612 | 0.121 | 1.183 | 0.591 | 2.091 | **0.021** | 1.094 | 0.775 |
| rs701492 | interaction | 1.023 | 0.941 | 1.228 | 0.500 | 0.910 | 0.756 | 0.766 | 0.397 |
| rs16858996 | interaction | 1.073 | 0.879 | 2.547 | 0.076 | 1.392 | 0.498 | 0.647 | 0.388 |
| rs17701824 | interaction | 0.803 | 0.428 | 1.247 | 0.418 | 1.326 | 0.305 | 0.852 | 0.563 |
|  |  |  |  |  |  |  |  |  |  |
