# Supplementary material for: Gender Differences in Associations of Glutamate Decarboxylase 1 Gene (GAD1) Variants with Panic Disorder
Source: PLoS One. 2012 May 25;7(5):e37651. doi: 10.1371/journal.pone.0037651 (PMC3360757; doi:10.1371/journal.pone.0037651)
Supplement: Table S4 — Gender-specific associations of GAD1 polymorphisms in samples with and without co-morbid agoraphobia. Analysis of gender subsamples was performed only for polymorphisms that displayed significant gender differences in the discovery case-control sample (see Table 1); rs4439928 did not meet the quality criteria in the replication sample and was therefore not included in the analysis of the combined sample. Nominally significant results are shown in bold. Abbreviations: AG, agoraphobia; D, discovery sample; OR, odds ratio; p, p-value; R, replication sample; SNP, single nucleotide polymorphism. (DOC) [file pone.0037651.s006.doc]

| ***SNP*** | ***Coefficient*** | ***D-AG*** | | ***D+AG*** | | ***D+AG+R*** | |
| --- | --- | --- | --- | --- | --- | --- | --- |
| ***rs ID*** |  | ***OR*** | ***p*** | ***OR*** | ***p*** | ***OR*** | ***p*** |
| rs1978340 | interaction | 0.515 | 0.189 | 1.275 | 0.475 | 1.459 | 0.079 |
| rs1978340 | female | 0.909 | 0.789 | 0.755 | 0.160 | 0.769 | **0.022** |
| rs1978340 | male | 0.468 | **0.033** | 1.039 | 0.891 | 1.122 | 0.527 |
| rs3791878 | interaction | 1.105 | 0.825 | 0.962 | 0.909 | 0.797 | 0.314 |
| rs3762555 | interaction | 1.1*10^-9^ | 0.999 | 6.4*10^8^ | 0.999 | 1.558 | 0.608 |
| rs3762555 | female | 1.8*10^9^ | 0.999 | 2.573 | 0.265 | 1.096 | 0.836 |
| rs3762555 | male | 2.049 | 0.564 | 1.6*10^12^ | 0.999 | 1.708 | 0.470 |
| rs3749034 | interaction | 0.497 | 0.336 | 0.611 | 0.423 | 1.04 | 0.921 |
| rs3749034 | female | 2.169 | 0.162 | 1.2 | 0.609 | 0.994 | 0.977 |
| rs3749034 | male | 1.077 | 0.875 | 0.733 | 0.535 | 1.034 | 0.922 |
| rs2270335 | interaction | 1.281 | 0.582 | 1.053 | 0.880 | 1.059 | 0.794 |
| rs2241165 | interaction | 0.563 | 0.257 | 0.444 | 0.054 | 0.889 | 0.631 |
| rs2241165 | female | 1.676 | 0.168 | 1.558 | **0.035** | 1.177 | 0.193 |
| rs2241165 | male | 0.943 | 0.865 | 0.691 | 0.313 | 1.046 | 0.833 |
| rs11542313 | interaction | 2.479 | 0.099 | 1.719 | 0.152 | 1.455 | 0.128 |
| rs3828275 | interaction | 0.921 | 0.876 | 0.624 | 0.251 | 0.674 | 0.111 |
| rs2058725 | interaction | 2.518 | 0.123 | 2.12 | 0.053 | 1.564 | 0.077 |
| rs701492 | interaction | 2.117 | 0.196 | 1.873 | 0.096 | 1.471 | 0.116 |
| rs16858996 | interaction | 0.314 | 0.165 | 0.609 | 0.470 | 0.504 | 0.218 |
| rs17701824 | interaction | 1.000 | 1.000 | 2.4*10^10^ | 0.999 | 0.942 | 0.851 |
| rs4439928 | interaction | 0.817 | 0.705 | 0.610 | 0.216 | -- | -- |
