# Supplementary material for: Gender Differences in Associations of Glutamate Decarboxylase 1 Gene (GAD1) Variants with Panic Disorder
Source: PLoS One. 2012 May 25;7(5):e37651. doi: 10.1371/journal.pone.0037651 (PMC3360757; doi:10.1371/journal.pone.0037651)
Supplement: Table S5 — Published gender-specific associations of GAD1 single nucleotide polymorphisms (SNP). (DOC) [file pone.0037651.s007.doc]

| ***SNP*** | ***Position*** | ***Alleles*** | ***Gender*** | ***Trait*** | ***Study*** |
| --- | --- | --- | --- | --- | --- |
| ***rs ID*** | ***basepair*** | ***minor/major*** |  |  |  |
| rs10432420 | 171660312 | G/A | female | schizophrenia | [1] |
| rs12185692 | 171670826 | A/C | female | depression | [2] |
| rs3791878 | 171672191 | T/G | male | schizophrenia | [3] |
| rs3749035 | 171672780 | A/C | female | schizophrenia | [1] |
| rs3749034 | 171673475 | A/G | female | schizophrenia | [4] |
| rs16823181/rs11542313 | 171678625 | T/C | female | schizophrenia | [1] |
| rs3791858 | 171689720 | C/A | male | schizophrenia | [3] |
| rs769407 | 171693708 | C/G | female | depression | [2] |
| rs3791850 | 171708100 | A/G | female | schizophrenia | [4] |

1. Straub RE, Lipska BK, Egan MF, Goldberg TE, Callicott JH, et al. (2007) Allelic variation in GAD1 (GAD67) is associated with schizophrenia and influences cortical function and gene expression. Mol Psychiatry 12: 854-869.

2. Utge S, Soronen P, Partonen T, Loukola A, Kronholm E, et al. (2010) A population-based association study of candidate genes for depression and sleep disturbance. Am J Med Genet B Neuropsychiatr Genet 153B: 468-476.

3. Du J, Duan S, Wang H, Chen W, Zhao X, et al. (2008) Comprehensive analysis of polymorphisms throughout GAD1 gene: a family-based association study in schizophrenia. J Neural Transm 115: 513-519.

4. Addington AM, Gornick M, Duckworth J, Sporn A, Gogtay N, et al. (2005) GAD1 (2q31.1), which encodes glutamic acid decarboxylase (GAD67), is associated with childhood-onset schizophrenia and cortical gray matter volume loss. Mol Psychiatry 10: 581-588.
