# Supplementary material for: Gender Differences in Associations of Glutamate Decarboxylase 1 Gene (GAD1) Variants with Panic Disorder
Source: PLoS One. 2012 May 25;7(5):e37651. doi: 10.1371/journal.pone.0037651 (PMC3360757; doi:10.1371/journal.pone.0037651)
Supplement: Table S6 — Associations of GAD1 polymorphisms with panic disorder in mixed-gender samples. rs4439928 did not meet the quality criteria in the replication sample and was therefore not included in the replication and combined mixed-gender sample analysis. Abbreviations: OR, odds ratio; p, p-value; SNP, single nucleotide polymorphism. (DOC) [file pone.0037651.s008.doc]

| ***SNP*** | ***Alleles*** | ***Discovery*** | | ***Replication*** | | ***Combined*** | |
| --- | --- | --- | --- | --- | --- | --- | --- |
| ***rs ID*** | ***minor/major*** | ***OR*** | ***p*** | ***OR*** | ***p*** | ***OR*** | ***p*** |
| rs1978340 | A/G | 1.252 | 0.119 | 1.074 | 0.596 | 1.154 | 0.144 |
| rs3791878 | T/G | 4.639 | 0.099 | 0.966 | 0.791 | 1.093 | 0.362 |
| rs3762555 | C/G | 0.844 | 0.255 | 1.172 | 0.254 | 1.005 | 0.958 |
| rs3749034 | A/G | 0.843 | 0.247 | 1.136 | 0.359 | 0.987 | 0.894 |
| rs2270335 | T/C | 0.835 | 0.214 | 1.098 | 0.496 | 0.964 | 0.710 |
| rs2241165 | C/T | 0.814 | 0.170 | 1.176 | 0.243 | 0.992 | 0.936 |
| rs11542313 | C/T | 0.878 | 0.317 | 0.955 | 0.714 | 0.918 | 0.342 |
| rs3828275 | T/C | 0.879 | 0.332 | 0.809 | 0.083 | 0.839 | 0.052 |
| rs2058725 | C/T | 0.938 | 0.661 | 1.076 | 0.600 | 1.007 | 0.945 |
| rs701492 | T/C | 1.115 | 0.462 | 1.096 | 0.477 | 1.105 | 0.303 |
| rs16858996 | G/A | 1.174 | 0.467 | 1.009 | 0.968 | 1.087 | 0.588 |
| rs17701824 | T/C | 0.936 | 0.616 | 1.074 | 0.555 | 1.009 | 0.919 |
| rs4439928 | G/A | 1.392 | 0.135 | -- | -- | -- | -- |
