# Supplementary material for: Gender Differences in Associations of Glutamate Decarboxylase 1 Gene (GAD1) Variants with Panic Disorder
Source: PLoS One. 2012 May 25;7(5):e37651. doi: 10.1371/journal.pone.0037651 (PMC3360757; doi:10.1371/journal.pone.0037651)
Supplement: Table S7 — Primer sequences used in this study. (DOC) [file pone.0037651.s009.doc]

| **SNP ID** | **Primary PCR Primer 1** | **Primary PCR Primer 2** | **Extend Primer** |
| --- | --- | --- | --- |
| rs3791878 | ACGTTGGATGAGGACCACGGTAAATACCAG | ACGTTGGATGTGTTGATGTCTGTGCGTGTC | GAATCACTGCCTACACG |
| rs17701824 | ACGTTGGATGCTGACCCTGAATGAGGAAGC | ACGTTGGATGGGGTGAGGTCACCTTTTTTC | ACAGGAGCTAGGGAGAT |
| rs2241165 | ACGTTGGATGTGCCCCGCCTCTCAGAGACA | ACGTTGGATGACTCTAAGCCCCCATCATAC | CCTCTCAGAGACACCGTTC |
| rs11542313 | ACGTTGGATGACTTACCGCAGATCTTGAGC | ACGTTGGATGCTCGTCCTAGCGTACGATAC | gaCAGTTTTCTGGTGCATCC |
| rs3749034 | ACGTTGGATGAAGCAGCTGGAGGTGACGC | ACGTTGGATGTTTCCTCTGCACAGCGCCCA | tggGACGCCGGGCAGATTAC |
| rs16858996 | ACGTTGGATGCATTTTTGCAACTGAGGGTC | ACGTTGGATGGGGGAGAAACTCTATATCAT | agGAGGGTCCAGGTTGATGT |
| rs769406 | ACGTTGGATGATGGGAATGCCTTTTCCCTC | ACGTTGGATGTCCCTCAATGAAATGGCCTG | gtCACTCCAGAACAAACCTCT |
| rs3828275 | ACGTTGGATGAGGATTTAGAGAGGATTCTG | ACGTTGGATGCACGGCTTTAAACCTGCATC | GAGAGGATTCTGATTCCTAGTA |
| rs701492 | ACGTTGGATGAATTATTGGAAAAGGAGGG | ACGTTGGATGGCTTTTTGCTGCTGCTAAAGT | tGGAGGGAAATTAAAATGAAGA |
| rs2058725 | ACGTTGGATGTTTGAGGGACAACGCCATTC | ACGTTGGATGTGCTCTACATGCGCTTTCTC | tCGCCATTCTGGGCTTTCATAAT |
| rs769390 | ACGTTGGATGTGTATGACTATGGCTTGTTG | ACGTTGGATGGAAAAAATGACCAGGGAAAC | tcTGACTATGGCTTGTTGCTTTAA |
| rs4668331 | ACGTTGGATGCAATCAATCAATGAGAAAGAC | ACGTTGGATGGTACCTGCAATTGTCTCCTG | GAGAAAGACAATAAACAAGTAGTT |
| rs4439928 | ACGTTGGATGCAAACCCATTCAGTCATGTA | ACGTTGGATGGGGAAATTCGTTGGGGTGAC | gtgATCATCATGATCAAGGTCTACA |
| rs2270335 | ACGTTGGATGTCACTGAGCGCTCCCCTGT | ACGTTGGATGGTCCAAGGCCAGTACAAGAC | CCCCTGTGCTCCTAGCC |
| rs3762555 | ACGTTGGATGAGAGAAATTCCCACCAGAGG | ACGTTGGATGTCGGGCGCTGGCGGAGAGA | GCCAAGAGCCCAGAGAC |
| rs12472230 | ACGTTGGATGGGAAAGGCAAAAGTCTAGGG | ACGTTGGATGCCCTTTGTACTGAACACCTC | ACAAAGCAGAGGTTGCCAAG |
| rs769395 | ACGTTGGATGAGGTCGATTCTCACCAACAG | ACGTTGGATGTCCAACAACTCCAGGAAAAC | TTCTCACCAACAGCATTTCCCT |
| rs769393 | ACGTTGGATGTCTTGGCTCTTGCTGGCTAC | ACGTTGGATGTTCCTTAAAGGTTCAGTTGC | GCTTCTTCTTTGATCAGTACTCA |
| rs1978340 | ACGTTGGATGTGTCCTGTCTACTCACCTTG | ACGTTGGATGTTGAAGGAGCGCCCAAACG | ACCTTGACTGACCACGTTTTAGGC |
